# The association between social support and depression among patients with vitiligo in China

**DOI:** 10.3389/fpsyg.2022.939845

**Published:** 2022-08-23

**Authors:** Xiaoying Ning, Yanfei Zhang, Wei Wang, Huling Yan

**Affiliations:** Department of Dermatology, The Second Affiliated Hospital of Xi’an Jiaotong University, Xi’an, China

**Keywords:** vitiligo, social support, depression, SDS, psychosocial, SSRS

## Abstract

Vitiligo is a common depigmenting skin disease with profound psychosocial impacts. Depression is one of the most common mental distress. Social support has a significant impact on the psychological status of patients. The aim of this study is to investigate the depression and social support status of patients with vitiligo in China, and further explore the correlation between social support and depression. The outpatients with vitiligo were investigated face to face with SDS (Self-rating depression scale) and SSRS (Social support rate scale). The mean SDS score of the patients was 44.05 ± 6.76, which was significantly higher than Chinese norms (*p* = 0.000). Female patients, unmarried, disease at rapid progressive stage and skin lesions at the exposed site had higher SDS scores (all *p* < 0.05). The scores of total social support, subjective support, objective support and support availability were lower than Chinese norms (all *p* < 0.01), and all were negatively correlated with SDS scores (all *p* < 0.001). In conclusion, low social support is one of the risk factors for depression in patients with vitiligo in China. More support and acceptance should be given to the patients.

## Introduction

Vitiligo is an acquired depigmenting disease caused by the destruction of melanocytes (Ezzedine et al., 2015). The prevalence of vitiligo is about 0.1–4% in the world. The exact incidence rate is difficult to estimate, due to different race, region and population (Sahni et al., 2011). It is estimated that about half of the patients occur before the age of 20, and 70–80% of the patients occur before the age of 30 (Sehgal and Srivastava, 2007). The etiology and pathogenesis of vitiligo are complex. Genetic factors, autoimmunity, oxidative stress and neuroendocrine factors are involved, but the specific mechanism is not clear (Frisoli et al., 2020). It is now considered that vitiligo is a psycho-dermatology disease (Simons et al., 2020). Although vitiligo will not bring serious health problems, it could cause significant cosmetic problems and serious psychological damage to patients (Simons et al., 2020). The psychological burden of this skin disease is relatively high (Ezzedine et al., 2021). Patients with vitiligo are more likely to suffer clinical depression or depressive symptoms (Lai et al., 2017).

Social support includes objective support, subjective support from all aspects of society and individual utilization of social support. It is an important factor affecting the adaptation of patients to chronic diseases (Shao et al., 2020). Studies have showed that social support is a protective factor for mental and physical health (Åvitsland et al., 2020; Khoury et al., 2021). In addition, studies have also mentioned the importance of social acceptance on patients’ quality of life (Ratajska et al., 2020; Sitjar-Suñer et al., 2020). Patients complain that they are socially intolerant and unacceptable because of their appearance, and they do not seem to receive the family or social support they deserve (Struck et al., 2020).

Based on the fact that the social support will affect patients’ psychological status, the purpose of this cross-sectional study was to investigate the social support and depressive symptom of patients with vitiligo in China, the outpatients with vitiligo were investigated face to face with Self-rating depression scale (SDS) and Social support rate scale (SSRS), and further analyze the impact of social support on patients’ depressive symptom.

## Materials and methods

### Ethical approval

This study and protocols were approved by the Institutional Review Board of the Xi’an Jiaotong University. And performed in accordance with the rules laid down in the Declaration of Helsinki. Informed consent has been obtained from all patients.

### Participants

The patients with vitiligo who visited the dermatology clinic of the Second Affiliated Hospital of Xi’an Jiaotong University from August 2020 to December 2021 were selected as the research object. The inclusion criteria were: (1) All patients were over 18 years old. (2) The diagnosis of vitiligo was made by two dermatologists and wood lamp (LeWitt and Kundu, 2021). (3) All patients had no previous or family history of mental illness, such as depression. Exclusion criteria were: (1) Patients were under 18 years old. (2) Patients with other skin diseases or serious systemic diseases may lead to depression, such as psoriasis, acne, alopecia areata and other cosmetic diseases.

### Data collection

All patients who met the inclusion criteria were evaluated by general information questionnaire, SDS and SSRS. Zung Self rating Depression Scale is an official instrument to measure depressive tendency, in the form of a self-report questionnaire (Zung, 1965). It includes 20 item Likert scales to explore psychological and physiological symptoms and is scored by the respondents according to the application mode of each item in the past week. SDS is widely used in rough screening, emotional state assessment, investigation, scientific research, etc., and can be used as an auxiliary tool for diagnosing potential cases, but not used for diagnosis. The Chinese version was used in our study, which has good reliability and validity (Tian et al., 2019). The SSRS was compiled by Xiao Shuiyuan according to China’s national conditions with good reliability and validity (Xiao, 1994). It includes 10 items in three dimensions: subjective support, objective support and support availability. The sum of the scores of 10 items is the total score. Higher than 44 points means that social support is at a high level, 23–44 points is at a medium level, and lower than 23 points is at a low level (Dai et al., 2016).

### Statistical analysis

The data were statistically analyzed by SPSS 18.0 statistical software. The mean ± standard deviation was used to describe the measurement data. The data were analyzed with single sample *t*-test, One way analysis of variance (ANOVA), two independent sample *t*-test and Pearson two-variable correlation analysis. *P*-values less than 0.05 was considered significant.

## Results

### Basic demographic data

As shown in Table 1, this cross-sectional study was conducted on 170 patients with vitiligo, including 87 males (51.2%) and 83 females (48.8%). The ratio of men to women was 1:1.05. The patients were mainly young and middle-aged, under the age of 60. More than half of the patients (55.88%) had college education or above. There were more married patients than singled patients (95 vs. 75), and nearly three-quarters of the patients had no family history of vitiligo. For severity of disease, 62.9% patients were mild. 123 patients (72.35%) had skin lesions on the exposed sites of the head, face, neck and hands. 75.3% patients with disease at progressive or rapid progressive stage.

**TABLE 1:** Basic data of patients with vitiligo.

Characteristics		N	%
Gender	Men	87	51.18
	Women	83	48.82
Age (years)	18–25	48	28.24
	25–40	65	38.24
	40–60	50	29.41
	>60	7	4.11
Marital status	Single	75	44.12
	Married	95	55.88
**Education level**			
	Junior middle school	12	7.06
	Junior high school	63	37.06
	Junior college and bachelor	82	48.24
	Master and doctor	13	7.64
**Occupation**			
	Civil servant	46	27.06
	Enterprise staff	71	41.76
	Students	28	16.47
	Other	25	14.71
**Family history**			
	Positive	43	25.29
	Negative	127	74.71
**Duration of disease (years)**			
	<1	52	30.59
	1–2	46	27.06
	2–5	35	20.59
	>5	37	21.76
**Lesions on the exposed sites**			
	Yes	123	72.35
	No	47	27.65
**Severity of disease**			
	Mild	107	62.94
	Moderate	38	22.35
	Moderate to severe	21	12.35
	Severe	4	2.36
**Stages**			
	Rapid progress	34	20.00
	Progressive	94	55.29
	Stable	42	24.71

### Self-rating depression scale score of patients with vitiligo

The mean SDS score of 170 patients with vitiligo in this survey was 44.05 ± 6.76, higher than the Chinese norms 41.88 ± 10.57 (Wang et al., 1986), the difference was statistically significant (*t* = 4.181, *p* = 0.000). As shown in Table 2, there were no significant differences in SDS scores among patients of different ages, occupations and educational levels (all *p* > 0.05). There was no significant difference in scores between patients with or without a family history of vitiligo (*p* > 0.05), the same as patients with different duration of disease and disease severity (all *p* > 0.05). For SDS score, it was found that the score of female patients was significantly higher than that of males (*t* = 4.83, *p* = 0.00), single patients higher than that of married patients (*t* = 3.683, *p* = 0.000). Lesions on the exposed sites had higher SDS score (*t* = 2.04, *p* = 0.043). The scores of patients at different disease stages varied significantly (*t* = 10.128, *p* = 0.000). Patients at rapid progress stage had higher SDS score than those in progressive and stable stage (*t* = 5.07, *p* = 0.000 and *t* = 6, 19, *p* = 0.000, respectively).

**TABLE 2:** Univariate analysis of patients with vitiligo in the score of SDS (mean ± SD).

Variables	SDS	*t/F*	*P*
**Gender**			
Female	46.46 ± 6.76	4.834	0.000
Male	41.75 ± 5.94		
**Age (years)**			
18–25	44.79 ± 6.65	0.756	0.520
25–40	43.06 ± 7.06		
40–60	44.52 ± 6.80		
>60	44.71 ± 3.82		
**Marital status**			
Single	46.12 ± 6.80	3.683	0.000
Married	42.41 ± 6.30		
**Education level**			
Junior middle school	43.50 ± 7.91	2.348	0.074
Junior high school	44.89 ± 6.66		
Junior college and bachelor	44.20 ± 6.18		
Master and doctor	39.54 ± 8.53		
**Occupation**			
Civil servant	44.04 ± 6.01	1.113	0.346
Enterprise staff	44.79 ± 6.91		
Students	44.07 ± 7.50		
Other	41.92 ± 6.73		
**Stages**			
Rapid progress	48.38 ± 4.02	10.128	0.000
Progressive	43.31 ± 6.88		
Stable	42.19 ± 6.90		
**Lesions on the exposed sites**			
Yes	44.63 ± 7.13	2.044	0.043
No	42.53 ± 5.47		
Family history			
Positive	45.37 ± 6.24	1.493	0.137
Negative	43.60 ± 6.89		
**Duration of disease (years)**			
<1	44.38 ± 6.76	0.433	0.730
1–2	43.46 ± 7.90		
2–5	44.91 ± 5.06		
>5	43.49 ± 6.77		
**Severity of disease**			
Mild	43.42 ± 7.13	1.650	0.180
Moderate	45.21 ± 5.31		
Moderate to severe	44.05 ± 6.76		
Severe	49.75 ± 6.95		

### Social support of patients with vitiligo

The total score of social support of 170 patients with vitiligo in this study was 39.22 ± 7.23, lower than the Chinese norms (Tan et al., 2021), the difference was statistically significant (*t* = 9.238, *p* = 0.000), indicating that the social support of patients with vitiligo was at a medium level. The objective support score was 22.45 ± 5.06, subjective support score was 9.25 ± 2.77 and support availability was 7.52 ± 1.73, all these scores were lower than the Chinese norms (Tan et al., 2021) (all *p* < 0.01) (see Table 3). As shown in Table 4, the correlation analysis between patients’ social support and SDS score showed that objective support, subjective support, support availability and the total score of social support were negatively correlated with patients’ SDS score (all *p* = 0.000).

**TABLE 3:** Comparison between social support scores in patients with vitiligo and Chinese norms (mean ± SD).

Variables	Patients	Chinese norms	*t*	*P*
Social support	39.22 ± 7.23	44.34 ± 8.38	9.238	0.000
Subjective support	22.45 ± 5.06	23.81 ± 4.75	3.512	0.001
Objective support	9.25 ± 2.77	12.68 ± 3.47	16.166	0.000
Support availability	7.52 ± 1.73	9.38 ± 2.40	13.956	0.000

**TABLE 4:** Correlation analysis between patients’ social support and SDS score.

Variables		SDS
Social support	R	–0.495
	*P*	0.000
Subjective support	R	–0.405
	*P*	0.000
Objective support	R	–0.363
	*P*	0.000
Support availability	R	–0.303
	*P*	0.000

## Discussion

The frequency of mental illness increased in patients with severe pigmented diseases (Dabas et al., 2020). Due to cosmetic defects, people with vitiligo often feel distracted and avoid social interaction, which will inevitably affect their mental health and may lead to stress-related disorders, depression, and even suicide. Study has shown that depression is common in patients with vitiligo (Sampogna et al., 2008). An Indian study assessed patients with vitiligo using the skindex-61 scale, which showed a high incidence of mental disorders, the most common was depression (Sarkarm et al., 2018). A meta-analysis review showed that the prevalence of depression in patients with vitiligo was high (Wang et al., 2018). The prevalence of depression in patients with vitiligo in Asia was much higher than that in Caucasus. The social stigma of vitiligo patients was more serious in Asia than in other regions (Wang et al., 2018). A two-way cohort study of population-based vitiligo and major depression disorder (MDD) found that patients with MDD had an increased risk of diagnosing vitiligo compared with those without MDD. The use of antidepressants has a protective effect on the risk of vitiligo. The risk of MDD patients who use antidepressants was significantly lower than that of MDD patients who do not use antidepressants (Vallerand et al., 2019). Consistent with the above findings, our study showed that the mean SDS score of vitiligo patients was significantly higher than that of the general population in China.

Female patients with vitiligo are more likely to suffer from depression than male patients. One possible explanation is that women have a high perception of skin color, which affects their perception of beauty. Also, in some cultural contexts, changes in skin appearance may affect marriage and family relationships. Vitiligo is considered as a defect in marriage (Elbuluk and Ezzedine, 2017). Also found in our study, the singled patients had higher SDS score. The singled patients are more likely to feel uncomfortable and embarrassed when getting along with others. They may be considered unclean, or rejected by their partners and deemed unfit for marriage for fear that vitiligo will be passed on to the next generation, putting them under enormous marital pressure and psychological burden. In addition, the social acceptance of single patients with vitiligo was significantly lower than that of married people (Elbuluk and Ezzedine, 2017). In our study, patients who have lesions on the exposed sites had higher SDS score. The higher the visibility of vitiligo, the more comments and discrimination patients may suffer in social communication, and their behavior of avoiding social interaction is also more obvious. Likewise, skin diseases, such as acne, which occur in highly visible areas, like the face, were associated with depression (Vallerand et al., 2018). Patients at rapid progressive stage had higher SDS score. The progression of the disease makes it easier for patients to feel fearful and helpless about the disease, leading to psychological problems such as anxiety, depression, and anger. Clinicians should actively evaluate the depressive symptoms/signs of patients with vitiligo and provide appropriate referrals to manage their mental symptoms accordingly. Future research should been done to determine the effect of antidepressants and other mental health treatments on the remission and recurrence of vitiligo and its mechanism.

Social support is very important for the change of healthy behavior. Good social support can increase the courage and confidence of individuals to deal with problems and get rid of difficulties, enable them to actively face difficulties, establish healthy behavior, and maintain good physical and mental health. Our results showed that the social support of patients with vitiligo was at a medium level, low social support and support utilization were the risk factors of depressive symptoms. The social acceptance of vitiligo was low, with various misconceptions and negative attitudes (Bidaki et al., 2018). A survey of the public’s attitude toward vitiligo found that 20% of people believed that vitiligo was an infectious disease, 33% did not know whether vitiligo was infectious, 23% believed that vitiligo was caused by poor hygiene habits, and more than half of people said they were reluctant to marry someone with vitiligo (Alghamdi et al., 2012). It was found that compared with the control group, patients with vitiligo received significantly less support from their social networks (Picardi et al., 2003). Clinicians involved in the care of patients with vitiligo should be aware of the close relationship with social support and depressive symptoms, and engage with mental health professionals when needed. Therefore, we should actively publicize the vitiligo related knowledge, improve the public’s awareness of vitiligo, and advocate the society to give more support and encouragement to patients with vitiligo, so as to promote the development of physical and mental health of patients with vitiligo.

This study also has some limitations: (1) The sample survey was conducted in only one hospital. (2) The sample size was limited, resulting in a small number of cases collected at certain ages, disease severity and stages. In the future, multi-center and large-sample studies should be carried out. (3) Location of patchy skin discoloration or changes to hair color was not explored in the analysis. (4) Only use the SDS to assess the depressive symptoms of patients with vitiligo, and more professional and detailed evaluation may be required in the future. 5. Lack of research on the effect of psychotherapy in patients with vitiligo.

## Conclusion

Our study shows that there is a significant relationship between social support and depressive symptom among patients with vitiligo in China. These results can provide reference for the clinical practice and support the use of social support as an intervention for vitiligo patients with depressive symptom. More support and acceptance should be given to the patients.

## Data availability statement

The original contributions presented in this study are included in the article/supplementary material, further inquiries can be directed to the corresponding author.

## Ethics statement

The studies involving human participants were reviewed and approved by the Institutional Review Board of the Xi’an Jiaotong University. The patients/participants provided their written informed consent to participate in this study.

## Author contributions

YZ and XN: writing – original draft and review and editing, software, formal analysis, investigation, and visualization. HY: supervision. WW: investigation. All authors contributed to the article and approved the submitted version.
